# Genomic Analysis of Differentiation between Soil Types Reveals Candidate Genes for Local Adaptation in *Arabidopsis lyrata*

**DOI:** 10.1371/journal.pone.0003183

**Published:** 2008-09-11

**Authors:** Thomas L. Turner, Eric J. von Wettberg, Sergey V. Nuzhdin

**Affiliations:** 1 Center for Population Biology, University of California Davis, Davis, California, United States of America; 2 Molecular and Computational Biology, University of Southern California, Los Angeles, California, United States of America; University of Texas Arlington, United States of America

## Abstract

Serpentine soil, which is naturally high in heavy metal content and has low calcium to magnesium ratios, comprises a difficult environment for most plants. An impressive number of species are endemic to serpentine, and a wide range of non-endemic plant taxa have been shown to be locally adapted to these soils. Locating genomic polymorphisms which are differentiated between serpentine and non-serpentine populations would provide candidate loci for serpentine adaptation. We have used the *Arabidopsis thaliana* tiling array, which has 2.85 million probes throughout the genome, to measure genetic differentiation between populations of *Arabidopsis lyrata* growing on granitic soils and those growing on serpentinic soils. The significant overrepresentation of genes involved in ion transport and other functions provides a starting point for investigating the molecular basis of adaptation to soil ion content, water retention, and other ecologically and economically important variables. One gene in particular, *calcium-exchanger 7*, appears to be an excellent candidate gene for adaptation to low Ca∶Mg ratio in *A. lyrata*.

## Introduction

Serpentine soil mosaics are a classic context for ecological adaptation [1], [2]. These widespread soils occur in small patches along fault lines where igneous rocks such as serpentinite are exposed. This environment is characterized by a suite of challenging abiotic factors such as low calcium-to-magnesium ratios, increased heavy metal concentrations, nutrient deficiency, and low moisture retention [3], leading to sharp transitions in abiotic conditions at the boundaries of serpentine patches [4]. The ecological community is differentiated along these boundaries as well: serpentine soils support an considerable array of endemic plant species, with serpentine specialists comprising 12.5% of native California plants, despite these soils comprising only 1% of the land area in the region [5]. Some widespread species are also found on serpentine, with several documented cases of local adaptation to soil conditions (e.g. *Collinsia* [6], *Cerastium* [7], and *Pinus* [8]). Populations of *Arabidopsis lyrata*, a perennial self incompatible crucifer, grow on serpentine outcroppings, interspersed with populations on other soil types such as granitic outcroppings and sand dunes [9]. Though information regarding local adaptation is lacking for *A. lyrata*, its proximity to the genetic model organism *A. thaliana* provides an opportunity to locate polymorphisms which are associated with the serpentine soil habitat. The role of these polymorphisms in serpentine adaptation, if any, can then be experimentally investigated.

By hybridizing genomic DNA from *A. lyrata* to *A. thaliana* Affymetrix tiling arrays, we can measure genetic differentiation between soil types at 2,853,369 probes throughout the *A. lyrata* genome. When DNA is hybridized to the array, probes which overlap a polymorphic SNP or indel will hybridize poorly in individuals with the mismatched allele [10]–[14]; when hybridization intensity is significantly different between populations, a differentiated polymorphism can be mapped to a specific location in the genome [15]–[17]. This method facilitates the discovery of adaptive variation in several complementary ways. First, we can discover candidate genes for adaptation to important environmental conditions, such as the low Ca∶Mg ratio which is characteristic of serpentine soil. In *A. thaliana* (which cannot normally survive on serpentine soils), an induced loss of function mutation in the calcium-proton antiporter *cax1* enhances survival on soils with a low Ca∶Mg ratio [18]: differentiation of natural variation at related genes in *A. lyrata* would provide clear candidate loci for adaptation to low Ca∶Mg ratio. Second, we can use the natural distribution of genetic variation between environments to investigate the functions of unannotated genes and non-coding features. If an uncharacterized locus consistently assorts with soil ion content, for example, then it can be hypothesized to interact with this environmental variable to determine fitness. Third, we can use genetic differentiation at genes with known function to form hypotheses about other environmental differences which may be important in nature. Although abiotic factors are thought to be the most important drivers of serpentine adaptation, if differentiation is also found in genes coding for anti-microbial compounds, anti-predatory compounds, or proteins which mediate competitive interactions, then parasitism, predation, or competition can be inferred to be important.

In the current work, we have hybridized genomic DNA from two serpentine and two granitic populations of *A. lyrata* to the *A. thaliana* tiling array. This has allowed us to locate many polymorphisms which are differentiated between soil types, including excellent candidates for adaptation to soil conditions. We do not mean to imply that selection is the only force which leads to differentiation between populations, as stochastic demographic forces may also lead to correlated distributions of genetic polymorphisms and environmental conditions, especially in this small sample of populations [19]–[21]. Indeed, there has long been a debate as to the relative importance of selection and demography in creating population differentiation [19], [22], [23]. This debate has only intensified in the genomic era, as it is now clear that selection can be rampant, and lead to adaptive change at loci across the genome [24]–[26]. Our paper is not intended to resolve this debate–rather, we provide a genomic portrait of differentiation which is necessary but not sufficient to determine which polymorphisms are influenced by spatially varying selection. This data can then inform functional investigation of candidate genes and processes.

## Results and Discussion

### Genomic analysis

**Figure 1:**
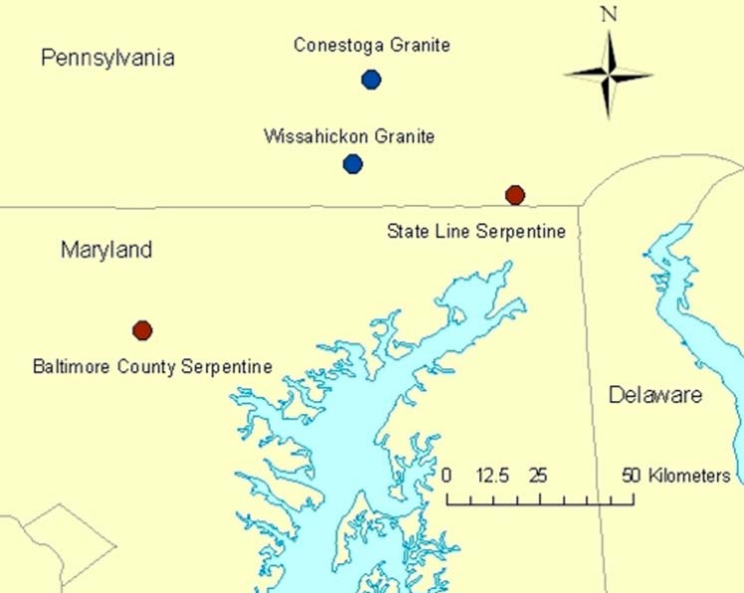
Map of collection locations.

**Figure 2:**
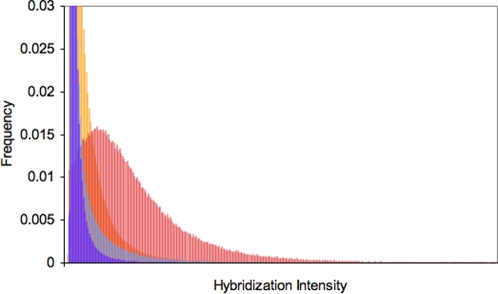
Distributions of hybridization intensities. Probes with a perfect match in the draft *A. lyrata* genome have the highest mean intensity (red), and the corresponding mismatches for these probes have the next highest mean (orange). For probes without a match in the draft genome, intensities are still higher at perfect match (grey) than mismatch (purple) probes. Though some of these probes might have a matching DNA sequence in our populations, but not in the reference genome, many of these probes are probably comparisons between a single mismatch and a double mismatch, yet are still providing valuable information. Note that extreme values are not shown for both axes.

To map differentiated polymorphisms between serpentine and granitic *A. lyrata* localities, DNA from three individuals from each of the four localities (figure 1) was fragmented, labelled, and hybridized to an *A. thaliana* tiling array (12 total arrays). On the array, each perfect match (PM) probe that matches the genome is adjacent to a mismatch (MM) probe, which has a mismatched base at the middle base pair. Comparison of normalized Σ(PM_i_) and Σ(MM_i_) intensities over the *i* chips for each probe indicates that the probes matching the draft *A lyrata* genome are the most sensitive markers of DNA differentiation, as expected (figure 2). For these 371,642 probes, the Σ(PM_i_)−Σ(MM_i_) is positive for 98.8% of probes. For the other 2,481,727 probes on the array, the Σ(PM_i_)−Σ(MM_i_) is positive at 65% of probes. This indicates that probes matching the draft genome are the most sensitive markers for detecting DNA differentiation, but also that the 2.48 million other unique probes contain useful information.

**Figure 3:**
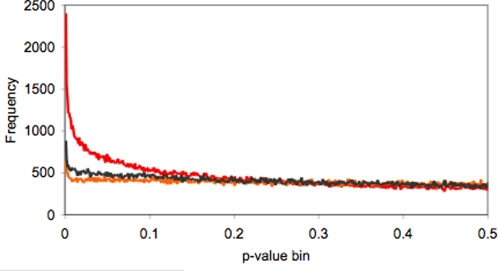
Histogram of *t*-test *p*-values between collection localities (bin size = 0.001). Three combinations of our four populations were tested: test 1 is the soil type comparison (blue line) = serp1+serp2 vs. granite1+granite2. For comparison, we also computed the other two combinations of the four localities: test 2 (red line) = serp1+granite1 vs. serp2+granite2; test 3 (green line) = serp2+granite1 vs. serp1+granite2).

To determine which probes overlapped differentiated polymorphisms, we computed a *t*-test *p*-value for between soil types at each array probe; the distribution of *p*-values for probes with a perfect match in the draft *A. lyrata* genome is shown in figure 3. There is a considerable excess of probes with low *p*-values: 2402 probes have *p*<0.001, whereas only 371 are expected by chance. For comparison, *t*-tests were computed between the other two possible combinations of our four collections. Dividing the samples along these additional axes revealed a small excess of probes with low *p*-values in both cases (figure 3), but substantially fewer than in the serpentine vs. granitic comparison, indicating that most differentiation between these samples assorts with soil type.

After correcting for multiple tests, 39 probes are significantly differentiated between soil types at a bonferroni *p*<0.05 (for this and all further analyses, the full 2.48 million probes were used; see Materials and Methods for details). Using a less conservative false discovery (FDR) rate criterion, 168 probes are significant at a FDR<0.01, and 751 probes are significant at a FDR<0.05. The 39 bonferroni significant probes overlap 33 genes, including two genes with multiple significant probes. When the FDR<0.01 probes are included, 112 genes overlap at least one significant probe; adding the FDR<0.05 set of probes increases this number to 455 genes including 81 genes with 2–12 significant probes each (Data Set S1). To locate additional differentiated genes, we used a sliding window analysis to determine if probes with low, but not individually significant FDR are clustered in the genome. Using permuted data to generate a null distribution, we mapped windows of 5, 10, and 20 probes that are significantly enriched for probes with FDR<0.30. Seventy-one small genomic regions were significantly differentiated between serpentine and granitic samples (windows with a permutation-based FDR<0.001 were considered significant, see methods). These regions are mostly a few kb in size (mean = 6.6 kb), with 11 regions larger than 10 kb and 3 larger than 30 kb. These 71 regions overlap 184 total genes, including 52 genes already significant from the analysis of individual probes.

The combined list of significantly differentiated loci (probes with FDR<0.05 and windows with FDR<0.001) includes 586 genes, 545 of which are annotated with at least one function in *A. thaliana* (based on gene ontology assignments, hereafter referred to as GO terms). When the probes with 0.01<FDR<0.05 are excluded to generate a more conservative list, the number of GO associated genes drops to 263. As noted in the introduction, additional data is required in order to determine how many of these genes are differentiated due to spatially varying selection. We can, however, use the annotations of differentiated loci to prioritize specific genes and molecular functions for further investigation. We find that the genes which are differentiated between soil conditions are associated with a non-random subset of biological functions (Data Set S1). Membrane proteins (*p*<0.001) and transporters (*p*<0.001) are very overrepresented among differentiated genes. GO terms which are obvious candidates for adaptation to soil salt and nutrient content include potassium ion transport (*p* = 0.006), cellular calcium ion homeostasis (*p* = 0.008), and cation transport (*p* = 0.03). These genes provide a starting point for understanding the molecular basis of adaptation to known environmental differences, such as low Ca∶Mg ratios, and also may indicate additional unappreciated environmental variables. An example of this second category is the differentiated gene *AT4G10380*, which responds to boron limitation in *A. thaliana* [27]. Differentiation of this gene motivates the investigation of the role of this important micronutrient in soil adaptation.

We also discovered suites of related genes that would not be obvious *a priori* candidates for soil adaptation: one of the most overrepresented GO terms is microtubule-based movement (*p*<0.001). Six genes with this function are differentiated, including a putative kinesin heavy chain and four genes which share a kinesin motor protein domain. It is possible that these loci interact with spatially varying environmental variables either directly (gene-by-environment interactions), or to compensate for deleterious side effects of adaptive change at other loci (gene-by-gene-by-environment interactions). An alternative, neutralist, explanation for the overrepresentation of GO terms among differentiated loci is that genes differ in polymorphism rates, and genes with more polymorphism are more likely to have some polymorphisms which are differentiated due to stochastic forces. It should be noted that the overrepresentation of GO terms is not specific to the granitic vs. serpentine population comparison. We also conducted GO tests between the other two combinations of our four populations (serp1+granite1 vs. serp2+granite2 and serp1+granite2 vs. serp2+granite1). In order to have the same power for all tests, the GO test of serpentine vs. granitic soils was re-computed using only the 751 significant probes, and compared to the probes with the lowest 751 *t*-test *p*-vales from the other two combinations of the data. For the significant probes, 33 GO terms are significant, while in both other combinations of the data, many different GO terms are significant (20 and 46 terms, respectively). Whether this is due to spatially varying selection on different process along different geographic axes, or simply variation in neutral differentiation rates remains to be determined.

### Genetic analysis

**Figure 4:**
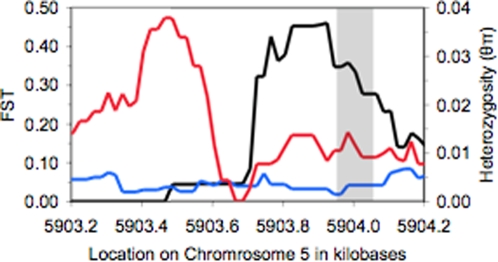
Sequencing of cax7 revealed a small region of high differentiation between soil types: 200 bp sliding windows of FST are shown in black. Serpentine populations have much less polymorphism in this region that granitic populations (200 bp sliding windows of heterozygosity are shown in blue for serpentine, red for granite). Vertical lines delineate a region of 70 bp where 8 of 23 amino acids are polymorphic in granitic populations and fixed in serpentine populations.

An induced null mutation in the calcium-proton antiporter *cax1* in *A. thaliana* is the only characterized mutation which enhances survival on soils with low Ca∶Mg ratios [18]. A related gene, *cax7* (*AT5G17860*), contains a significantly differentiated probe in our analysis. This gene, though functionally distinct from *cax1* [28], is therefore an excellent candidate for adaptation to low Ca∶Mg ratios in *A. lyrata*. We sequenced 1.3 Kb of this gene in a larger sample of plants from the four *A. lyrata* collections and found a small region of high differentiation towards the 5′ end of the coding sequence (figure 4, table 1). Patterns of polymorphisms at this locus are quite different on the different soils: individuals from granitic soils have high nucleotide diversity, many intermediate frequency amino acid polymorphisms, and significantly positive values of Tajima's D, Fu and Li's D, and Fu and Li's F (*p*<0.05; [29], [30]. Plants from serpentine soils, on the contrary, have low levels of polymorphism and negative (though non-significant) values of the above statistics; Fay and Wu's H is −3.31 for serpentine plants, indicating many high frequency derived mutations on these soils. One 70 bp region of *cax7* exemplifies these patterns: in this region, 8 of 23 amino acids are polymorphic in granitic collections, perhaps due to ongoing gene flow from serpentine plants, while all are fixed on serpentine, consistent with strong purifying selection against such gene flow. Fine-scale studies of this gene involving many individuals and environmental conditions can now be conducted to determine the environmental and population-genetic forces influencing this variation, and the phenotypic consequences of the polymorphic alleles.

**Table 1:** Analysis of sequence data, with individuals grouped by soil type.

		bp	n	F_ST_	θπ	Taj D	Fu&Li D	Fu&Li F	Fay&Wu H
AT5G17860 (cax7)	granite	1326	24	0.45	0.0144	2.239	2.045	2.611	4.587
	serpentine	1326	20		0.0036	−0.966	−0.670	−0.970	−3.305
AT4G35290	granite	2167	22	0.11	0.0002	−0.312	0.413	0.227	1.750
	serpentine	2167	22		0.0002	−1.720	−1.547	−1.862	1.403

bp = base pairs sequenced, n = chromosomes sequenced, θπ = heterozygosity.

We also sequenced 2.2 kb of *AT4G35290*, one of three putative ligand-gated ion channels with a significant probe, contributing to the significant overrepresentation of genes involved in cellular calcium ion homeostasis. Unlike *cax7*, we found similar levels of polymorphism at this locus in the four populations, with no skews in the allele frequency spectrum (θ_π_ = 0.0006 and 0.0012 for granitic populations, 0.0007 and 0.0008 for serpentine populations). Although this gene contained a significant (FDR = 0.04) probe, this probe did not overlap a differentiated polymorphism and is therefore a false discovery. This gene was in our least significant category, 0.05>FDR>0.01, which is expected to include some false discoveries. Including these probes also increases the sensitivity of the analysis, however: the significant probe in *cax7* had a FDR = 0.011, and would have also been excluded had we only used a threshold FDR of 0.01. Despite reduced power when probes with 0.01<FDR<0.05 are excluded, 20 GO terms remain significant, including cellular calcium ion homeostasis. The conservative and comprehensive lists of significant probes, regions, genes and GO terms are available as supplementary data (Data Set S1).

### Copy number variation

Many of our significant regions appear to be large differentiated duplications and deletions (copy number variants, or CNVs). By comparing the intensity of probes in each significant region to an expected distribution generated using probes from all significant regions, we established that 15 of the 71 differentiated regions are likely CNVs (at a threshold *p*<0.001, see methods); this includes 7/11 regions larger than 10 kb, and all three regions larger than 30 kb. As an example, a 33.9 kb significantly differentiated region on chromosome 4 spans 11 genes (map coordinates refer to the *A. thaliana* assembly). The average intensity of the 99 probes in this region in granitic plants is 1.2 (median normalized, where the median intensity = 1.0), while the serpentine plants have an average intensity of 2.6, which appears to indicate that there is one copy of this region in most granitic individuals and two or three copies in most serpentine individuals. Other cases appear to be differentiated deletions: there are 21 probes which overlap the gene *AT5G27100*, one of three significantly differentiated ligand-gated ion channels. The average intensity of these probes is 0.986 on serpentine, near the median of all probes, and 0.167 on granite. PCR amplification of this gene from an expanded sample of 48 individuals validated the presence of a deletion with higher frequency in granitic populations: deletion frequencies are 0.79 and 0.65 for the granitic collections and 0.24 and 0.38 for the serpentine collections. The existence of CNVs whose frequencies are associated with environmental conditions, including large duplications of many genes, provides an excellent opportunity to study the influence of selection in the initial stages of gene duplication [31]. A similar study of clinal adaptation in *Drosophila melanogaster* also found a considerable contribution of copy number variation to population differentiation, including large variants that affected multiple genes [17]. These two experiments, together with adaptive CNVs in humans [32], indicate that copy number variation is a prolific source of adaptive polymorphism in natural populations. Interestingly, changes in gene copy number have also recently been shown to provide heavy metal tolerance in the closely related species *A. halleri* [33].

### Conclusions

Between the four populations used in this study, many more loci are differentiated between serpentine and granitic populations than along other geographic axes. The neutralist interpretation of this result is that these populations are more distantly related due to recent or historical migration patterns. However, recent genomic studies of polymorphism, differentiation, and divergence clearly indicate the pervasive effects of selection [25], [26], [34]–[36]. It is therefore very difficult to infer whether the observed patterns of differentiation are due to patterns of migration, rampant selection, or both effects [24]. We believe that the most powerful approach will be combining “from the genes up” approaches like the current work with more traditional approaches “from the traits down”. For example, reciprocal transplants of individuals between populations can directly measure fitness, which can then be associated with the differentiated polymorphisms detected here.

## Materials and Methods

### Sample collection

Seeds were collected at two serpentine sites and two granitic sites in close proximity (figure 1). Although historically there were 26 serpentinic outcroppings in an arc from Virginia to Staten Island, New York, nearly half have been destroyed by landuse changes in greater Philadelphia and Baltimore; of the remaining outcroppings, two are large (1000 acres or greater) and largely protected, while the others are small (2–10 acres) and altered by successional changes [37]. Although a few of the smaller barrens do have small *A. lyrata* populations (frequently fewer than 100 individuals, EVW unpublished data), we restricted our analyses to the two patches which we estimate contained over 10,000 individuals. The two non-serpentinic populations sampled are the only ones approximately equidistant to the two serpentinic populations that we could find based on herbaria records (R. Latham, unpublished) and our own search; the other five sites with herbaria collections on non-serpentinic substrates we visited had disappeared due to ecological succession or human alteration. The serpentinic sites are part of the Nature Conservancy's state line serpentine barren complex in Chester County, Pa, and Soldier's Delight Natural Environment Area, Baltimore County, Md. Granitic sites were located at the Lock 12 recreation area on the Susquehanna river (York County, Pa), and Lancaster County Park (Lancaster County, Pa).

### Microarray hybridization

DNA was extracted from three plants from each of the four populations using Qiagen plant mini-kits and amplified using rolling-circle amplification with the Qiagen Repli-G kit. After phenol-chloroform clean up and ethanol precipitation, 10 µg of DNA from each sample was fragmented with DNase following Turner *et al.* 2008. To fragment DNA, we created a master mix of DNase I (Promega), One-Phor-All buffer (Amersham Biosciences), and Acetylated BSA (Invitrogen); the amounts added per sample were 4 µl 10X One-Phor-All, 0.14 µl Acetylated BSA, and approximately 0.085 µl of DNase per ug of DNA. The precise amount of DNase added per sample depends on the batch of enzyme: our goal was to calibrate the reaction to obtain fragmented DNA of approximately 50 bp, with low variance in fragment size. Fragmentation was performed in a MJ Research PTC-200 thermocycler at 37 C for 16 min, 99 C for 15 min, and 12 C for 15 min. Samples with similar intensities and fragment sizes (approximate fragment size = 50 bp) were labelled with a master mix of Biotin-N6-ddATP (Enzo) and RTdT enzyme (Promega) to each sample. RTdT was diluted from 30 U/µl to 15 U/µl enzyme before use by mixing RTdT enzyme, RTdT 5X buffer, and water at a ratio of 5∶1∶4. For each sample to be labelled, 2 µl of Biotin and 3 µl of RTdT were added to the master mix. Labelling was accomplished in a thermocycler at 37 C for 90 min, 99 C for 15 min, and 12 C for 5 min. Microarrays were hybridized at the Affymetrix core facility in the UC Davis Genome Center using standard conditions for this array.

### Microarray analysis

Using NCBI megablast, we determined that 2,853,369 array probes have a single perfect match in the TAIR7 *A. thaliana* reference genome and retained these probes for analysis (that is, we exclude probes with more than one exact match in the reference genome). We spatially normalized array intensities at these probes by dividing each array into 1600 subarrays of 64 by 64 probes, and divided the log(intensity) of each probe by the median log(intensity) of the local 64×64 probe window. We further normalized these values using quantile normalization in R [38]. Of these 2.85 million probes, 371,642 (13%) had a perfect match in a preliminary 4× draft of the *A. lyrata* genome (kindly provided by DOE-JG's Community Sequencing Program [proposal coordinated by Detlef Weigel, MPI Tübingen]).

To find differentiated polymorphisms, *t*-tests were computed for each probe. We first analyzed only the probes with a match to *A. lyrata*, and corrected these *p*-values for 371,642 multiple tests. We used three significance thresholds in order to have inclusive and conservative lists of differentiated probes: a bonferroni-corrected *p*<0.05, and two false discovery rate (FDR) thresholds: FDR<0.01 and FDR<0.05 [39]. Next, we computed *t*-test *p*-values for the remaining 2,481,727 probes, correcting these values for the greater number of multiple tests, and discovered additional probes significant at the three thresholds. We combined these two sets of significant probes to generate a list of differentiated loci at the three levels of stringency, which are available in data set S1. Note that FDR calculations assume a uniform distribution of truly null features (*p*-value * number of multiple tests / *p*-value rank), and are therefore estimates. These estimates are based on technical, and not biological, false discovery: a probe with low specificity may truly detect differentiation, but not at the expected location.

To increase our power to find differentiated loci, we used a sliding window analysis to determine if probes with low FDR are clustered in the genome. For this analysis, we only used probes with a single perfect match in the draft *A. lyrata* genome. There were 5,338 probes with FDR<0.30 (*p*<0.003): these probes have high individual rates of false discovery, but can be used as markers to find regions of the genome that are enriched for differentiated probes. Using permuted chromosomes to generate a null distribution, we mapped windows of 5, 10, and 20 probes that are significantly enriched for candidate markers. Each window recorded the average probe FDR, and compared this value to the permuted chromosomes. Probes with FDR>0.30 were considered to have an FDR = 1.00, as this was previously found to make the analysis more conservative [17]. Because FDR estimates for windows were found to be less accurate than FDR estimates for individual probes [17], we considered windows significant only if they had FDR<0.001 when compared to the permuted chromosomes.

### Differentiation of functional categories

Using the TAIR7 annotation of the *A. lyrata* reference genome, we determined which genes overlapped our differentiated probes and windows; a gene was considered to overlap a differentiated locus if any portion of its transcript overlaps a significant probe or window. Significance of gene ontology (GO) terms among our significant genes was determined in two complementary ways: a binomial sampling test, and a permutation test (GO terms are a controlled vocabulary which classifies genes based on all known functional associations determined computationally or experimentally). First, we compared the observed proportion of significant genes in each GO term to an expected number, which is the binomial sampling probability of sampling an equal or larger number of genes in each category given the number of significant genes in the genome. For the permutation test, we simulated our discovery procedure by randomly sampling probes and genomic regions of the same size and number as our significant probes and regions. For example, in the primary analysis presented below, there are 751 significant probes and 71 significant regions. To generate a null distribution of genes for this analysis, we sampled this same number of probes and regions (of an equal size distribution), and determined which genes overlap the random sample. We considered a GO term to be significantly overrepresented if the observed sample has more genes in the given term than 5% of 500 simulated data sets, and less than 5% probability of occurring by chance in the binomial sampling test.

### Copy number variants

If serpentine and granitic populations are equidistant to the reference genome, then any given differentiated probe should have higher intensity on serpentine half the time, and higher intensity on granite half the time. In some significant regions, however, all probes are more intense on one soil type, consistent with differentiated copy number variants (CNVs). Differentiated regions are considered candidate CNVs if the ratio of mean hybridization intensity for that region is more extreme than expected. We extracted the raw (unnormalized) intensity values for all probes in each significant region, and computed a mean intensity for serpentine and granitic populations. These means were minimally normalized by dividing by the median intensity for all probes for each soil type, and the ratio of the normalized means was used as the test metric. An expected distribution of ratios was created by randomly sampling the number of probes in the test region from the set of probes from all significant regions. Regions were considered candidate CNVs if their ratio was more extreme than 9,990/10,000 random sets (*p*<0.001). This permutation controls for many possible confounding effects. For example, if all derived SNPs were found on serpentine, then granitic populations would have higher average intensities in significant regions; because our permutation samples only from significant regions, the candidate CNV are significantly beyond any such effect.

### Sequencing and PCR

Three loci were amplified via long PCR using Phusion polymerase (Finnzymes), and two were directly sequenced. Population genetic analysis of sequenced loci was done in DNAsp [40]; see table 1 for summary statistics. The third locus, *AT5G27100*, is polymorphically deleted. The presence/absence of this gene was investigated using primers within the deletion as mapped by the arrays. These primers amplified a fragment in 11/12 and 9/12 individuals in the serpentine1 and serpentine2 populations, but only 3/12 and 5/12 individuals in the granitic1 and granitic2 populations; 24 individuals were typed twice with complete reproducibility. Deletion frequency was calculated assuming Hardy-Weinburg equilibrium, as this is a dominant marker. Primers are available from the authors upon request.

## Supporting Information

Data Set S1 Complete lists of differentiated probes, regions, genes, and GO terms at multiple significance threshold. (Note that this is a large data set formatted as an .xls file with multiple spreadsheets. We have converted the initial sheet, of GO terms into a .pdf, but cannot easily submit the entire data set as .pdf.)
(0.03 MB PDF)
