# Supplementary material for: Genomic Analysis of Differentiation between Soil Types Reveals Candidate Genes for Local Adaptation in Arabidopsis lyrata
Source: PLoS One. 2008 Sep 11;3(9):e3183. doi: 10.1371/journal.pone.0003183 (PMC2527522; doi:10.1371/journal.pone.0003183)
Supplement: Data Set S1 — Complete lists of differentiated probes, regions, genes, and GO terms at multiple significance threshold. (Note that this is a large data set formatted as an .xls file with multiple spreadsheets. We have converted the initial sheet, of GO terms into a .pdf, but cannot easily submit the entire data set as .pdf.) (0.03 MB PDF) [file pone.0003183.s001.pdf]

Data set 1. Significant GO terms.
n = number of sig. genes, k = number of genes in term, p = p-value

GO term	n	k	p
lactose_catabolic_process,_using_glucoside_3-dehydrogenase	4	14	0.0001775
lactose_catabolic_process_via_UDP-galactose	4	15	0.00023039
kinase_activity	45	1342	0.00029211
transporter_activity	42	1233	0.00033885
ATPase_activity	10	136	0.00038664
beta-galactosidase_activity	4	18	0.00045648
lactose_catabolic_process	4	19	0.00055817
beta-galactosidase_complex	4	20	0.00067499
microtubule-based_movement	6	56	0.00086951
integral_to_membrane	18	399	0.00096243
regulation_of_shoot_development	2	3	0.00161064
cytokinin_receptor_activity	2	3	0.00161064
calcium:sodium_antiporter_activity	2	4	0.00282708
protein_amino_acid_phosphorylation	31	933	0.0028554
1-phosphatidylinositol-4-phosphate_5-kinase_activity	3	15	0.00324695
transport	49	1691	0.00340742
nucleotide_binding	37	1189	0.00347684
membrane	39	1276	0.00368451
plasma_membrane	15	366	0.00585629
microtubule_associated_complex	5	59	0.00619888
regulation_of_seed_germination	2	6	0.00620115
potassium_ion_transmembrane_transporter_activity	3	19	0.00623533
intracellular_ligand-gated_ion_channel_activity	3	20	0.00717056
cellular_calcium_ion_homeostasis	3	21	0.00818449
regulation_of_stomatal_movement	3	22	0.00927853
metabolic_process	19	536	0.00934106
microtubule_motor_activity	5	67	0.01034457
potassium_ion_transport	4	45	0.0118877
starch_metabolic_process	2	9	0.01343227
other_membranes	149	6557	0.01418754
transferase_activity	51	1924	0.01447431
stomatal_movement	2	10	0.01637497
defense_response,_incompatible_interaction	2	10	0.01637497
COPII_vesicle_coat	2	10	0.01637497
nuclear_pore	3	28	0.01758247
cation:cation_antiporter_activity	2	11	0.01956551
inward_rectifier_potassium_channel_activity	2	11	0.01956551
ATP_binding	22	703	0.02008987
hydrolase_activity	64	2574	0.02284657
trans-Golgi_network	2	12	0.02299336
alkaloid_biosynthetic_process	2	13	0.02664833
calmodulin-dependent_protein_kinase_activity	3	33	0.02684225
cation_transport	5	89	0.03053634
endoplasmic_reticulum	10	262	0.03326204
strictosidine_synthase_activity	2	15	0.03460028
protein_histidine_kinase_activity	2	15	0.03460028
base-excision_repair	2	16	0.03887833
response_to_oxidative_stress	7	162	0.03985703
inositol_or_phosphatidylinositol_kinase_activity	2	17	0.04334562
cellular_protein_metabolic_process	2	17	0.04334562
cytokinin_mediated_signaling	3	40	0.04337804
biosynthetic_process	5	100	0.04627508

FAD_binding	2	18	0.04799337
sucrose_catabolic_process,_using_beta-fructofuranosidase	2	18	0.04799337

GO terms with only one gene, where that gene is significant:

embryonic_root_morphogenesis	1	1	0.01911819
pollination	1	1	0.01911819
cellular_sodium_ion_homeostasis	1	1	0.01911819
cycloartenol_synthase_activity	1	1	0.01911819
acetate_metabolic_process	1	1	0.01911819
meiotic_chromosome_separation	1	1	0.01911819
SUMO-specific_protease_activity	1	1	0.01911819
calcium-dependent_protein_serine/threonine_kinase_activity	1	1	0.01911819
mitotic_chromosome_condensation	1	1	0.01911819
acquisition_of_desiccation_tolerance	1	1	0.01911819
tRNA_(5-methylaminomethyl-2-thiouridylate)-methyltransferase_ac	1	1	0.01911819
chloroplast_isoamylase_complex	1	1	0.01911819
nodulation	1	1	0.01911819
voltage-gated_calcium_channel_activity	1	1	0.01911819
positive_regulation_of_meiosis	1	1	0.01911819
ATPase_activator_activity	1	1	0.01911819
response_to_boron	1	1	0.01911819
structural_constituent_of_nuclear_pore	1	1	0.01911819
positive_regulation_of_sister_chromatid_cohesion	1	1	0.01911819
GTP_cyclohydrolase_I_activity	1	1	0.01911819
3-oxoacyl-[acyl-carrier-protein]_reductase_activity	1	1	0.01911819

excluding probes with FDR<0.01:

**GO term**	**n**	**k**	**p**
ATPase_activity	8	136	4.9869E-05
microtubule-based_movement	4	56	0.00200573
defense_response,_incompatible_interaction	2	10	0.0040646
COPII_vesicle_coat	2	10	0.0040646
trans-Golgi_network	2	12	0.00578191
alkaloid_biosynthetic_process	2	13	0.00674441
strictosidine_synthase_activity	2	15	0.0088704
metabolic_process	11	536	0.01271632
binding	15	843	0.01352415
intracellular_ligand-gated_ion_channel_activity	2	20	0.0152973
cellular_calcium_ion_homeostasis	2	21	0.01676325
regulation_of_stomatal_movement	2	22	0.01828656
microtubule_associated_complex	3	59	0.01829578
positive_regulation_of_flower_development	2	25	0.02318889
microtubule_motor_activity	3	67	0.02539297
protein_modification_process	3	70	0.0283836
nucleotide_binding	18	1189	0.03093273
cell_death	2	30	0.03239956
calmodulin-dependent_protein_kinase_activity	2	33	0.03850235
Golgi_apparatus	5	201	0.04126942

GO terms with only one gene, where that gene is significant:

cycloartenol_synthase_activity	1	1	0.00927192
SUMO-specific_protease_activity	1	1	0.00927192
calcium-dependent_protein_serine/threonine_kinase_activity	1	1	0.00927192

tRNA_(5-methylaminomethyl-2-thiouridylate)-methyltransferase_ac	1	1	0.00927192
nodulation	1	1	0.00927192
positive_regulation_of_meiosis	1	1	0.00927192
ATPase_activator_activity	1	1	0.00927192
response_to_boron	1	1	0.00927192
3-oxoacyl-[acyl-carrier-protein]_reductase_activity	1	1	0.00927192
